# Penetrating globe injury during rhinoplasty surgery; A case report

**DOI:** 10.1016/j.jpra.2023.10.011

**Published:** 2023-10-28

**Authors:** Ghafarian Sadegh, Sheikhghomi Sima, Bakhshaee Mehdi

**Affiliations:** a Assistant Professor of ophthalmology department. Farabi Eye Hospital, Tehran University of Medical Sciences, Tehran, Iran; b Assistant Professor of Ophthalmology department. Madani hospital, Alborz University of Medical Sciences, Karaj, Iran; c Full Professor of Otorhinolaryngology Department. Sinus and surgical Endoscopic Research Center, Mashhad University of Medical Siciences, Mashhad, Iran

**Keywords:** Rhinoplasty, Penetrating injury, Eye, Cataract

## Abstract

Complications of the rhinoplasty surgery may involve the orbital contents due to the anatomical proximity to the surgical site. In this article, we report an inadvertent complication of the rhinoplasty in a 32-year-old woman who presented to our clinic with reduced vision of her left eye following the surgery. With diagnosis of a penetrating globe injury, the patient underwent an anterior segment surgery with general anesthesia which included both corneal laceration repair and lensectomy. We suggest that surgeons consider using protective eye shields for their patients during these surgeries depending on their experience and technique.

## Introduction

Complications of the rhinoplasty surgery may involve the orbital contents due to the anatomical proximity to the surgical site. There are reports of orbital walls violence by the osteotomes with subsequent orbital infections or hemorrhages. There are also some reports of central retinal artery occlusion and total blindness due to the retrobulbar hemorrhages or periocular injections in these surgeries.^1^, ^2^, ^3^, ^4^, ^5^, ^6^, ^7^, ^8^ In this article, we report an inadvertent complication of surgery in a young woman of reduced vision after an esthetic rhinoplasty.

### Case presentation

**Figure 1:**
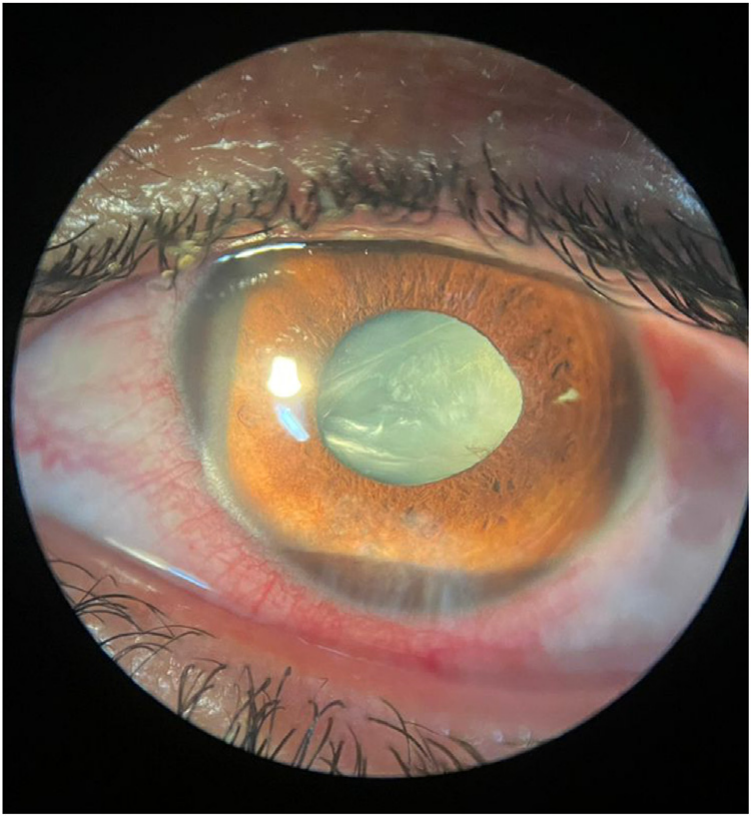
Broad beam slit lamp examination of the left eye shows a paracentral faint corneal opacity, pupil irregularity, iris hole in 3 o'clock and traumatic cataract.

**Figure 2:**
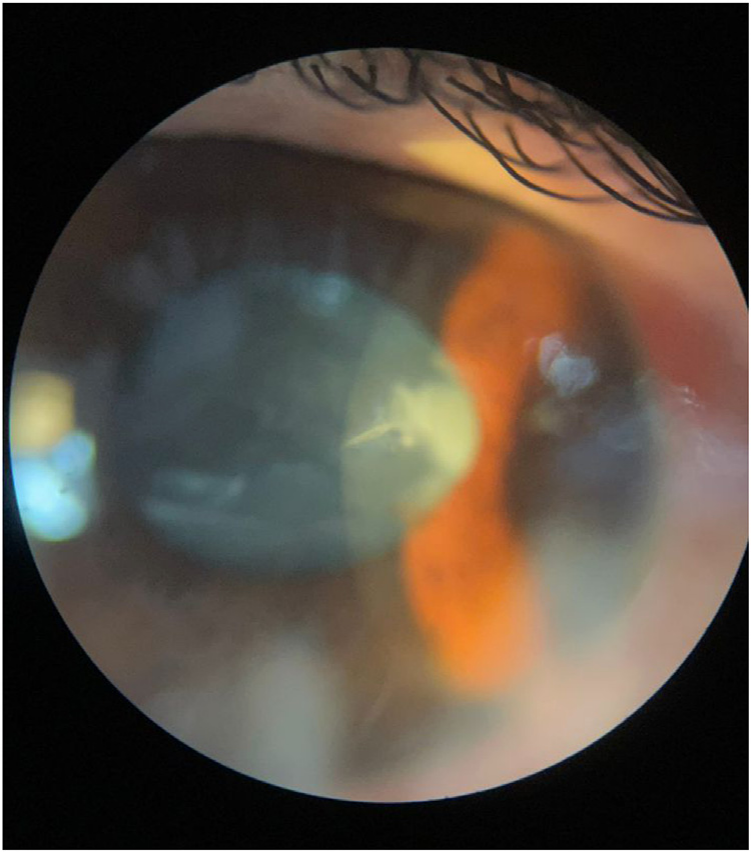
Narrow beam slit lamp examination with a more magnification and focus on the cornea, shows a linear 0.8 mm full thickness corneal laceration.

A 32-year-old woman presented to our clinic with reduced vision of her left eye which she noticed from 2 days ago, immediately after her cosmetic rhinoplasty. The patient denied any previous history of ocular or systemic diseases. On gross examination, a bilateral periocular ecchymosis was obvious due to the recent nasal surgery. The visual acuity of her left eye was hand motion and the right one was 10/10 (by the Snellen chart scale). The conjunctiva of her left eye was hyperemic and on microscopic examination, a paracentral linear 1 mm full thickness corneal laceration was detected accompanied with a small iris disruption behind the corneal laceration. The crystalline lens was partially white and opaque especially behind the iris defect. The anterior chamber depth was mildly shallower than the other side (Figure 1, Figure 2). An orbital CT scan was done and revealed no intra ocular foreign bodies. In a phone call contact with the patient's surgeon, her surgeon mentioned that he had realized no adverse event during that surgery but noticed the left eye at the end of the surgery which had turned to a hyperemic state.

**Figure 3:**
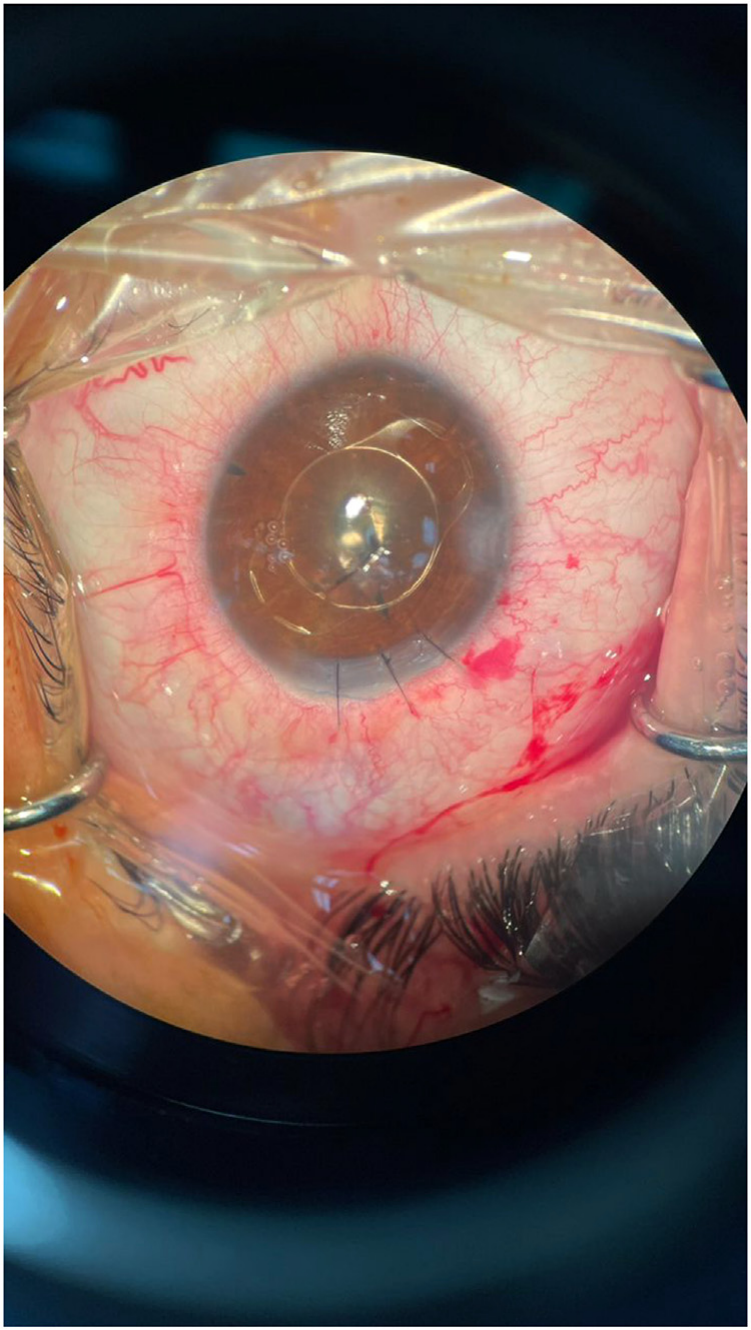
Immediately post-surgical view of the left eye shows repaired corneal laceration, anterior chamber iris fixed artisan IOL, and inferior peripheral iridotomy.

With diagnosis of a penetrating globe injury, the patient underwent an anterior segment surgery with general anesthesia which included both corneal laceration repair and lensectomy. Due to the associated traumatic posterior capsular rupture, anterior vitrectomy with limbal approach was also done and then an anterior chamber intra ocular lens (IOL) was placed thorough a superior scleral tunnel (Figure 3). Topical betamethasone and chloramphenicol was prescribed for the patient. Fortunately, the post-op examinations comprising the retinal examination showed no further damage of the posterior segment. The patient vision improved to 1/10 one week after the surgery.

## Discussion

Penetrating globe injury by the surgical instruments is not a never event in the facial plastic surgeries or outpatient interventions.^9^^,^^10^ Corneoscleral tissues pose a thickness less than 1 mm, and are vulnerable to be easily penetrated by the sharp needles or metal tools. In order to find out the mechanism of the injury, we arranged a detailed but friendly interview with the patient's surgeon. The surgeon stated that the rhinoplasty was open approached with local infiltration of 4cc lidocaine and 1/200,000 adrenalin around and inside the nose. A lateral osteotomy by 2 mm osteotome and dorsal hump removal by 14 mm osteotome and medial osteotomy by curve 4 mm osteotome was done. The tip plasty suturing was done with 5.0 polydioxanone(PDS) sutures. And the skin sutures was done with 6.0 nylon sutures. The surgeon couldn't clearly remember any tool slippage or collision during the surgery. However, we concurred that with attention to the small wound size and relatively deep penetrance into the eye, a needle would be the most probable offending instrument compared to the other sharp instruments including osteotomes or blades which are used in these surgeries. A much larger corneal wound would be expected by the deep penetrance of a blade or osteotome which goes far into the posterior lens capsule.

Surgical interventions near to the eyes require safety measures to prevent compromising these delicate tissues. In any event noted by the surgeon during surgery or by the patient after recovery, which an eye injury is suspected, the surgeon should call an ophthalmology specialist as soon as possible to reduce further consequences including infections or permanent visual loss. Klein et al. in 2012 introduced a new osteotome with protector and internal guide to reduce the probability of the escaping of the instrument during the surgery in order to prevent the surrounding tissues injuries.^11^ Nevertheless, this major complication about the eyes can be prevented much more cost-effectively by applying protective eye shields. Sterile protective eye shields are very cheap and a good barrier against misdirected sharp instruments. As far as we know, this is the first time that such a complication is described following the rhinoplasty. We considered it necessary to report this case to emphasize the importance of the protection of the eyes in such operations.

## Conclusion

Patient safety and reducing surgical risks are important rules of the surgical interventions including the rhinoplasty surgeries. We suggest that surgeons consider using protective eye shields for their patients during these surgeries depending on their experience and technique.

## Funding

None.

## Conflict of Interest

The authors declare that they have NO affiliations with or involvement in any organization or entity with any financial interest in the subject matter or materials discussed in this manuscript.
